# Electrophysiological Basis for Early Repolarization Syndrome

**DOI:** 10.3389/fcvm.2018.00161

**Published:** 2018-11-06

**Authors:** Rubén Casado Arroyo, Juan Sieira, Maciej Kubala, Decebal Gabriel Latcu, Shigo Maeda, Pedro Brugada

**Affiliations:** ^1^Department of Cardiology, Erasme University Hospital, Université Libre de Bruxelles, Brussels, Belgium; ^2^Heart Rhythm Management Centre, Universitair Ziekenhuis Brussel, Vrije Universiteit Brussel, Brussels, Belgium; ^3^Department of Cardiology, Centre Hospitalier Universitaire, Amiens, France; ^4^Department of Cardiology, Centre Hospitalier Princesse Grace, Monaco, Monaco; ^5^Advanced Arrhythmia Research, Tokyo Medical and Dental University, Tokyo, Japan

**Keywords:** early repolarization, idiopathic ventricular fibrillation, mapping, sudden cardiac death, Brugada syndrome, ventricular fibrillation

## Abstract

During last centuries, Early Repolarization pattern has been interpreted as an ECG manifestation not linked to serious cardiovascular events. This view has been challenged on the basis of sporadic clinical observations that linked the J-wave with ventricular arrhythmias and sudden cardiac death. The particular role of this characteristic pattern in initiating ventricular fibrillation has been sustained by clinical descriptions of a marked and consistent J-wave elevation preceding the onset of the ventricular arrhythmia. Until now, Early Repolarization syndrome patients have been evaluated using ECG and theorizing different interpretations of the findings. Nonetheless, ECG analysis is not able to reveal all depolarization and repolarization properties and the explanation for this clinical events. Recent studies have characterized the epicardial substrate in these patients on the basis of high-resolution data, in an effort to provide insights into the substrate properties that support arrhythmogenicity in these patients. An overview for the current evidence supporting different theories explaining Early Repolarization Syndrome is provided in this review. Finally, future developments in the field directed toward individualized treatment strategies are examined.

## Introduction

The concept early repolarization (ER) points out a J-point elevation and terminal QRS abnormalities that might have a relative high prevalence in the population. In the last two decades, it has been proposed that early repolarization pattern (ERP) may be associated with an increased risk of ventricular fibrillation (1–4).

When ERP is associated with ventricular tachycardia or ventricular fibrillation in the absence of organic heart disease, ERP is referred to as early repolarization syndrome (ERS). Some studies evaluated different parameters to distinguish benign ERP from the malignant type (ERS), based on its electrocardiographic appearance (3, 5–10). The evaluation of ERP has been difficult in the past due to the absence of a clear definition. It is because of this problem that a high variability in the incidence of ERP has been described.

## Epidemiology

ER pattern is significantly more common in blacks than in Caucasians. ER pattern seems to be more common in Aboriginal Australians than in Caucasian Australians (11). In the general population, the prevalence of an ER pattern in the lateral and/or inferior leads with a J point elevation of ≥ 0.1 mV ranges between 1b and 24%, and between 0b.6 and 6.4% for J point elevation of > 0.2 mV (11–13). These population-based and case control studies have provided some clinical evidence for an increased risk of suffering sudden cardiac death and life-threatening arrhythmic events in this population of patients presenting an ER pattern, particularly in inferior and infero-lateral leads (4, 10, 14).

Several epidemiological studies have tried to evaluate the risk of sudden cardiac death related to ER. A study from Finland evaluated ER in more than 10,000 patients. They described that the inferior ER pattern of 0.1 mV was present in 3.5% and lateral in 2.4% of the study population. J-Point Elevation in inferior or lateral leads was associated with death from cardiac arrhythmias. Interestingly, it was not the case in patients with J-point elevation in both inferior and lateral leads (*p* = ns) (2). Another study of more than 29000 patients in the USA evaluated resting ambulatory ECG. They found that J waves or other common patterns of ST segment elevation was not associated to cardiovascular death (11). A recent meta-analysis has described a modest increase risk in arrhythmic death 1.70 (95% CI: 1.19–2.42; *p* = 0.003) and no significative risk in cardiac death or non-cardiac death (13). Rosso et al. have calculated that an ER pattern in a young patient from 35 to 45 years would increase the probability of suffering an episode of VF from 3.4 to 11 in 100,000 patients (15). Other authors have argued that ER would be a marker or vulnerability more than a disease by itself. Some epidemiological studies have proposed that ER would increase the risk of VF in the context of myocardial ischemia (16). Future studies will clarify the clinical implications of ERP in a population without history of cardiac arrhythmias.

## Electrocardiographic pattern

The presence of J waves in the electrocardiogram (ECG) have previously been reported in cases of healthy individuals, particularly in young males, black individuals and athletes. Prominent J waves have been described in hypercalcemia, hypothermia and ischemia (17–21).

For decades, an early ERP, consisting of a J point elevation, a slur or notch of the terminal part of the QRS with and without an ST segment elevation, was considered as benign (22, 23).

The benign nature of this pattern was challenged in 2001 on the basis of experimental laboratory data (in coronary-perfused wedge preparations) showing that this ECG morphology is linked to the development of polymorphic ventricular tachycardia and ventricular fibrillation (19, 24–26). The clinical validation of this hypothesis was provided less than a decade later (1, 15, 27).

Sometimes, the J wave can be so tall and broad to mimic an ST segment elevation. In humans, the normal J wave often appears as a J point elevation, with part of the J wave hidden inside the QRS. A horizontal/descending ST-segment morphology has been associated with an increased arrhythmic risk in the population with inferolateral ER (3, 28–30).

The evaluation of the ST segment in cases of ER is complex and sometimes suited to different interpretations. Also, there is a lack of consensus regarding whether only the leads with a J wave should be evaluated, or if only the revelation of a compatible morphology in a single lead is enough to make a diagnosis. In this situation, some research projects have started to evaluate the T wave and its relation to the R wave. Recently, a study has evaluated the characteristics of the T wave of 92 malignant inferolateral ER syndrome versus a group of 247 controls (30). The study has revealed that ERS patients present a lower amplitude of T waves, a lower T/R ratio in lead II or V5 and also al prolonged QTc interval. The data revealed that the combination of ERS and a QTc in the upper normal limit had an ominous prognosis.

Before considering the J-wave amplitude as a marker of risk, some limitations should be acknowledge. First, as demonstrated in a population study of more than 10,000 individuals, the prevalence in the general population of a J-point elevation in the inferior leads > 0.2 mV is very low (0.3%) (2). Second, J-point elevation is dynamic. 18.3% of the population with >0.1 mV didn't present this pattern in the follow up (1). Also, it has been shown that J wave increases preceding episodes of ventricular arrhythmias (1).

Some studies have tried to identify other risk factors associated with SCD (14). Pause dependent augmentation of the J wave has been proposed as a possible marker of risk (31). Twenty patients out of forty idiopathic ventricular fibrillation presented a pause dependent augmentation of the J wave. This characteristic had a lower sensitivity (55%) but a high specificity (100%) for ERS (31). New prospective observational data is needed to confirm these findings. Other authors have identified different ST-morphology variations linked with different phenotypes of ER (3). Descending or horizontal ST segment after J-point patients present an increased risk of sudden cardiac death. Also, ascending ST segments is not associated with sudden cardiac death (32).

## Diagnosis of ERS

A great number of discussions regarding the diagnosis and identification relative to ER pattern have taken place in the past. The diagnosis criteria, based on consensus papers on ERP, have gradually changed from the initial focus on ST-segment elevation toward the abnormalities of the terminal QRS (slurring or notching), J wave and the evaluation of the T wave (10, 19, 32–34).

For that reason, an expert consensus report focused on the definition of ER stated that to diagnose an ER pattern, the peak of an end QRS notch and/or the onset of an end QRS slur be designated as Jp. This point should exceed 0.1 mV in ≥2 contiguous inferior and/or lateral leads of a standard 12-lead ECG. The QRS duration should be less than 120 ms measured in leads without a notch or slur (10).

In 2009, a consensus defined the ER ECG pattern as “J-point elevation and rapidly upsloping or normal ST segment” as “a normal variant” (35). Six years later, another consensus defined ERP as a slur or an end-QRS notch on the downslope of a prominent R wave (10). The slur/notch should be above the baseline and the QRS duration should be <120 ms. A peak of the J wave of 0.1 mV in >2 contiguous leads of the 12-lead EC (excluding leads V1–V3).

In 2016 Based on the existence of higher incidence of some specific patterns in the ECG of patients who have suffered idiopathic VF, a statement proposed two criteria that increase the risk of presenting an episode of idiopathic ventricular fibrillation. J waves in II, III, aVF (inferior leads) and a descending/horizontal pattern of ST following the J point (36).

Lastly, in 2017, an international consensus document added some ECG criteria for the diagnosis of ER including a threshold of 0.2 mV for the amplitude of the J wave in two inferior or lateral ECG leads and also the existence of dynamic changes of the J-point (Shanghai ERS Score). In this context, a benign pattern has been identified that is characterized by an upsloping ST segment after the J point (37). The Shanghai ERS diagnosis Score presented in Table 1 is based on literature data and expert opinion. Due to the lack of large scale data or randomized controlled studies, rigorous weighted coefficients are lacking (34). However, the utilization of the scale can be regarded as a tool to orientate the clinician. Future studies will test this scale before using it in clinical practice.

**Table 1:** Proposed Shanghai Score System for diagnosis of early repolarization syndrome.

	**Points**
**I**. **Clinical History**	
Unexplained cardiac arrest, documented ventricular fibrillation or polymorphic ventricular tachycardia Suspected arrhythmic syncope Syncope of unclear mechanism/unclear etiology	3 2 1
**II**. **Twelve-Lead ECG**
Pattern A: ER ≥ 0.2 mV in ≥ 2 inferior and/or lateral ECG leads with horizontal/ descending ST segment. Pattern B: Dynamic changes in J-point elevation (≥0.1 mV) in ≥ 2 inferior and/or lateral ECG leads. Pattern C: ≥ 0.1 mV J-point elevation in at least 2 inferior and/or lateral ECG leads.	2 1.5 1
**III**. **Ambulatory ECG Monitoring**	
Short-coupled premature ventricular contractions with R on ascending limb or peak of T wave	2
**IV**. **Family History**	
Relative with definite ERS ≥2 first-degree relatives with a II.A. ECG pattern First-degree relative with a II.A. ECG pattern Unexplained sudden cardiac death ,45 years in a first- or second-degree relative	2 2 1 0.5
**V**. **Genetic Test Result**	
Probable pathogenic ERS susceptibility mutation	0.5
**Score (requires at least 1 ECG finding)**
≥ 5 points: Probable/ definite early repolarization syndrome 3–4.5 points: Possible early repolarization syndrome <3 points: Nondiagnostic

As a resume, ERS is diagnosed in patients who present “ER in the inferior and/or lateral leads presenting with aborted cardiac arrest, documented VF, or polymorphic VT” (15, 32).

ER is identified if all these criteria are met “(a) There is an end-QRS notch or slur on the downslope of a prominent R-wave. If there is a notch, it should lie entirely above the baseline. The onset of a slur must also be above the baseline; (b) the peak of the notch or J wave ≥0.1 mV in ≥2 contiguous leads of the 12-lead ECG, excluding leads V1–V3; and (c) QRS duration <120 ms” (15, 32).

Due to the difficult diagnosis of this syndrome, the differential diagnosis has a major importance. The differential diagnosis of ERP is presented in Table 2.

**Table 2:** Differential diagnosis of early repolarization pattern.

Metabolic disorders: Hypothermia, hyperthermia, hypocalcemia, hyperpotassemia Hypertensive heart disease Athlete's heart Myocardial ischemia (e.g., anteroseptal acute myocardial infarction) Thymoma Aortic dissection Arrhythmogenic right ventricular cardiomyopathy Takotsubo cardiomyopathy Intracerebral bleeding, acute cerebrovascular accident Pericardial disease, Myocardial tumor, and Myocarditis Chagas disease Cocaine intoxication

## Differences and similarities between brugada syndrome and ERS

Notwithstanding some difficulties to make a diagnosis of ERS, some publications have tried to differentiate both entities (38–41). The region affected of both entities appears to be different, RVOT in BrS vs. inferior or lateral left ventricle in ERS (42). Both entities exhibit different incidence of late potentials in signal-averaged ECGs.

In BrS, 60% of patients present late potentials vs. 7% in the case of ERS (43). The effect of sodium channel blockers is different in both entities, the elevation of the ST segment is higher in BrS than in ERS (44). The incidence of other arrhythmias is higher in BrS than in ERS (45). Lastly, some articles have described diverse structural alterations in BrS that are not present in ERS (46). Table 3 presents some differences between both entities.

**Table 3:** Differences between ERS and BrS.

	**BrS**	**ERS**
Region most involved	RVOT	Inferior LV wall
Leads affected	V_1_–V_3_, V5, V6, II, III, aVF (inferior and lateral repolarization cases)	II, III, aVF, V_4_, V_5_, V_6_; I, aVL
Global Incidence	Asia BrS > ERS	Europe BrS = ERS (not confirmed)
Incidence of late potential in signal- averaged ECG	Higher	Lower
Prevalence of atrial fibrillation	Higher	Unknown
Effect of sodium channel blockers on surface ECG	Increased J-wave	Reduced J-wave
Structural changes, including mild fibrosis and reduced expression of Cx43 in RVOT	Higher in severe forms of the syndrome	Unknown

## Genetics

Variants in 7 genes have been associated with both ER pattern and ERS (47–49). Variants in ABCC9 and KCNJ8, responsible for the pore forming and ATP-sensing subunits of the IK-ATP channel, have been reported in patients with ERS (12, 49, 50). These findings are in the same direction of the experimental models showing that IK-ATP activation can generate an ER pattern in canine ventricular wedge preparations. Loss-of-function variations in subunits of the cardiac L-type calcium channel (CACNA1C, CACNB2, and CACNA2D1) and the sodium channel (SCN5A and SCN10A) have been also reported linked to ERS (51–53).

It is important to note that only a fraction of identified variants have been evaluated using functional expression studies to clarify causality and also pathogenesis. A few have been studied in native or induced pluripotent stem cell derived from ERS patients. The limitation of the genetic test interpretation is based mainly in the lack of biological or functional validation (54). Table 4 shows the genes that have been associated with ER patterns and ERS.

**Table 4:** Gene defects associated with the early repolarization syndrome (ERS).

	**Locus**	**Gene**	**Ion channel**	**Percent of probands**
ERS1	12*p*11.23	KCNJ8	↑ I_K−ATP_	<1%
ERS2	12*p*13.3	CACNA1C	↓ I_Ca_	4.1%
ERS3	10*p*12.33	CACNB2b	↓ I_Ca_	8.3%
ERS4	7*q*21.11	CACNA2D1	↓ I_Ca_	4.1%
ERS5	12*p*12.1	ABCC9	↑I_K−ATP_	<1%
ERS6	3*p*21	SCN5A	↓ I_Na_	<1%
ERS7	3*p*22.2	SCN10A	↓ I_Na_	<1%

## Pathophysiologic evidence and animals models

**Figure 1:**
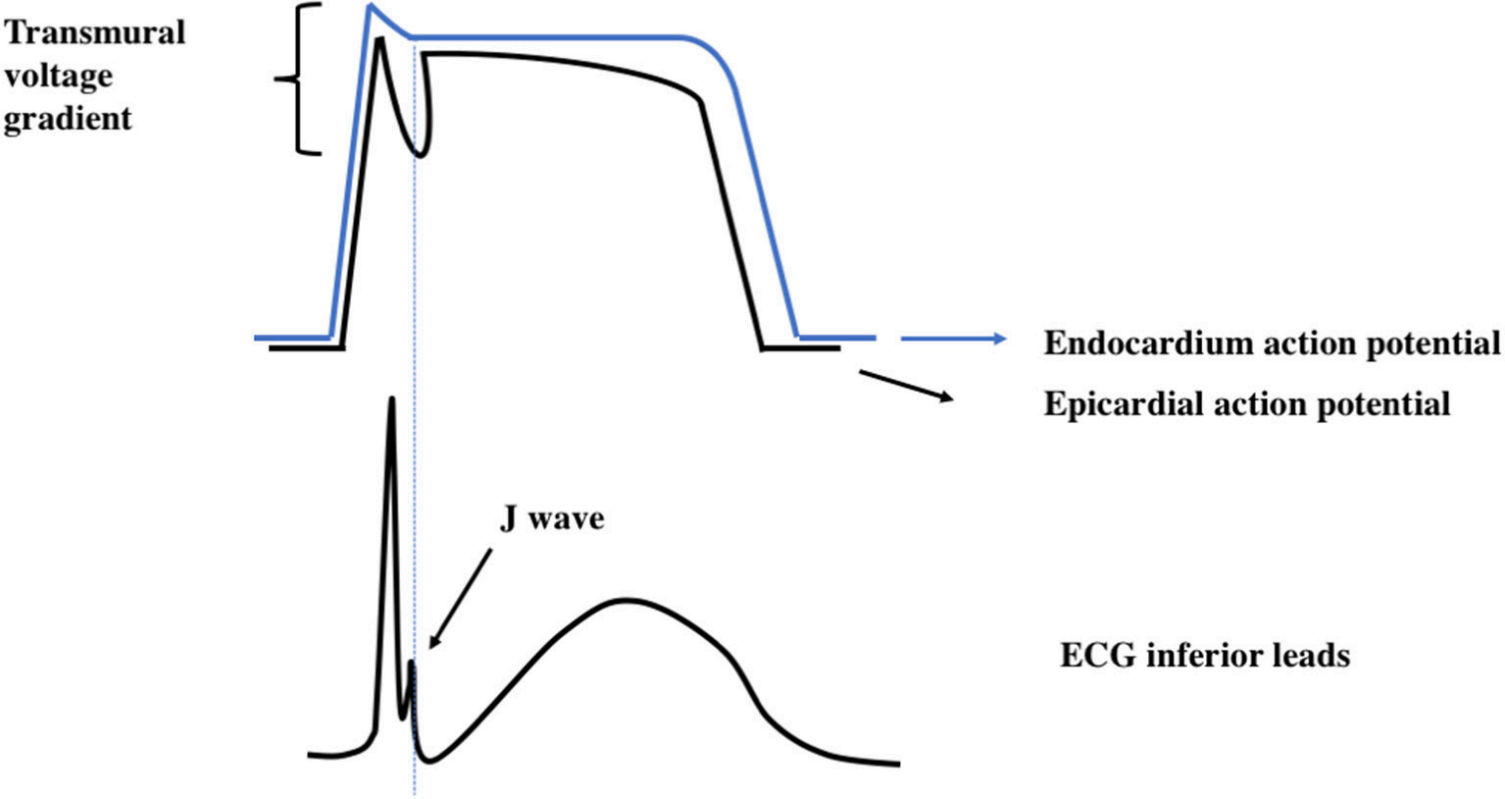
Proposed electrophysiological mechanisms of J waves. The upper panel shows the possible origin of the voltage gradient. These transmural differences of action potential are supposed to generate the ERP.

The pathophysiologic basis of the ER pattern is currently not entirely understood. A predominant theory states that J-point elevation appears in the context of a marked increase phase 1 notch of the epicardial action potential in relation to that of the endocardium. The consequence is an enhancement of ventricular transmural voltage gradient (Figure 1), which is illustrated as J-point elevation (55). Regarding the ionic mechanisms underlying an increased transmural voltage gradient, a marked increase in the epicardial action potential notch has been linked to a higher epicardial potassium current relative to the endocardium (55). Other ionic currents, including sodium, calcium and potassium-ATP dependent channels have also been described as involved in the repolarization variability in ER (26).

ER patients may also present a dynamic J-point variation. It has been described that this elevation is more obvious during period of bradycardia (56). The justification of this finding is that during episodes of increased vagal tone, the potassium currents (IK-ATP and IKACh) are increased and also there is a slow restoration of the Ito current (56). It should be note that in ERS, bradycardia mediated J-point elevation is more pronounced during episodes of high vagal tone than normal human beings.

Regarding the mechanism of ventricular arrhythmias, it seems that the dispersion of repolarization associated with ER enhances susceptibility to phase 2 reentry arrhythmias (55). As a consequence, a premature ventricular complex would interact with an predisposed ventricular substrate to trigger ventricular arrhythmias (57).

Recently, in an experimental model of canine ventricular preparation, an increment in vagal tone increase J-point elevation and induces phase 2 reentry. In addition, the authors showed that the intrinsic potassium current (Ito) was higher in the inferior wall of the left ventricle that could justify an increase sensitivity of this region for ventricular arrhythmias (58).

## Non invasive mapping in patients suffering ER syndrome

A recent publication has analyzed the electrophysiological substrate in 29 patients, 17 with the malignant form of ER syndrome and compared to 7 normal individuals (59). Body-surface ECG potentials were obtained simultaneously from 256 electrodes. Later, the patients underwent a CT scan with ECG gating to obtain the epicardial geometry and the electrode positions. The EP mapping data were evaluated for electrogram repolarization (measured by recovery time and activation recovery interval, the epicardial dispersion of repolarization was calculated based on the previous values), conduction (measured by activation time and activation duration) and morphology (J wave on local epicardial electrogram).

Epicardial J-wave was observed in EGMs from all ERS patients and in none of the controls. The study revealed in the ERS population, that the distribution of J waves was not localized to the inferior or lateral wall of the left ventricle, pointing that the substrate might not be limited to a specific region of the heart. The distribution of the epicardial J-wave was heterogeneous. Twenty seven percentage of the patients presented J-wave in the anterior wall, sixty five percentage in the lateral wall and seventy nine percentage in the inferior wall. There was an absence of fractionated electrograms. Also, there was not any data pointing to a delay activation in this group of ERS patients.

The data also showed that action potential duration in areas with J waves was shorter than the control group. In addition, the shortening of action potential duration was heterogeneous within the ventricle. The result was the creation of sharp repolarization gradients in comparison with controls. This characteristic is presented as the pathophysiologic basis of ERS and a differentiation with BrS. In BsS patients, a prolongation in the activation recovery interval has also been described. The latter has been studied also using ECGi (60). In BsS patients, both steep dispersion of repolarization and slow discontinuous conduction were present in the right ventricular outflow tract (60).

As a resume, this study has evaluated a cohort of ERS patients using noninvasive ECGi mapping, the arrhythmogenic abnormal substrate has been characterized by a heterogeneous shortening of the action potential duration and as a result, creation of steep repolarization gradients (59). Both mechanism could provide a substrate for re-entrant arrhythmias. These findings are different to those of BrS, where also a delay in ventricular activation has been described (60).

## Clinical implications

Although in the last two decades, a great number of articles have improved our understanding of ERS, there is an important knowledge gap regarding the pathophysiology, the clinical manifestations and management of ERS. ICD implantation is recommended for secondary prevention of SCD. In the field of primary prevention, ICD has been suggested by some authors as an option in case of “high risk” pattern, strong and unequivocal family history of SCD (37). It should be noted that the implantation of an ICD in the primary prevention setting is not free of complications (inappropriate shocks, infections). In this situation, the benefit of an implantation should be balanced against the risk of the complications. A clear definition of the syndrome and the risk of SCD in the primary prevention setting should be clarified before promoting the implantation of an ICD.

## Future directions

There are a lot of areas of uncertainty regarding the diagnosis, epidemiology, biological substrate, associations, prognosis and treatment of ERS. First, to avoid confusions regarding the diagnosis of the disease, ERS diagnosis should be based on prevalent and relevant variant patterns. Second, in order to identify the groups at risk and define the triggers, prospective studies should be performed in populations at risk. Third, due to the fact that the biological substrate is not completely understood, and the mechanism of ventricular arrhythmias are not fully elucidated, genetics and research in basic research would help to clarify the factors that promote arrhythmogenesis. Forth, there is still a lack of data to quantify predictive values and the number needed to treat in primary prevention. Fifth, the etiologic fraction of this pattern is probably still low and should be assessed. Sixth, due to the previous statements, an effective cost-effective treatment or preventive therapy are lacking. Large scale registries analyzing different cost-effective approaches will be necessary to deliver the best therapy for the population.

## Conclusion

ER is a frequent ECG characteristic in the general population. In a very small number of cases, ER is the unique apparent cause of SCD in and individual or family. Also, a complex genetic pattern favors the idea that ER is probably a disease-modifying factor than a standalone disease.

Therefore, proper identification of ER high risk patterns is critical to improve assessment and prevention. More research is needed to better understand the electrophysiological basis and clinical significance, prognosis and prevention of ER. The design of algorithms to integrate the stratification of risk of ER is a key topic for future research in the field of cardiac arrhythmias.

## Author contributions

RC: conception or design of the work, drafting the work, provide approval for publication of the content. JS, MK, DL, SM, PB: reviewers of the article and critical corrections, providing approval for publication of the content.

### Conflict of interest statement

The authors declare that the research was conducted in the absence of any commercial or financial relationships that could be construed as a potential conflict of interest.
